# Sacubitril/valsartan can improve the cardiac function in heart failure patients with a history of cancer: An observational study

**DOI:** 10.1097/MD.0000000000037613

**Published:** 2024-03-22

**Authors:** Zhulu Chen, Chuan Zhang, Yuxi Zhu, Diansa Gao, Min Mao, Zhong Zuo

**Affiliations:** a Department of Cardiology, The First Affiliated Hospital of Chongqing Medical University, Chongqing, China; b Department of Oncology, The First Affiliated Hospital of Chongqing Medical University, Chongqing, China.

**Keywords:** cancer, heart failure, HFmrEF, HfpEF, HfrEF, Sacubitril/Valsartan

## Abstract

Sacubitril/Valsartan, the combination of angiotensin receptor inhibitor and neprilysin inhibitor, is now becoming the class 1 recommendation for HFrEF. Some studies have shown the positive effect of Sacubitril/Valsartan on HFrEF cancer patients, while there is devoid of evidence about the effect of this drug in aged cancer patients with HFmrEF and HFpEF. By searching the patients with a diagnosis of both cancer and Heart failure (HF) over 65, the patients who had received treatment with Sacubitril/Valsartan were selected as the candidates for Sacubitril/Valsartan group, and the patients who had received conventional HF therapy without Sacubitril/Valsartan were chosen as the control group. Data were collected for up to 9 months. We filtered 38 patients and 50 patients valid for Sacubitril/Valsartan group and control group, respectively. After initiation of heart failure management, our study found a better cardiac condition in Sacubitril/Valsartan group, having better LVEF, LVFS, NT-proBNP in 3rd, 6th, 9th month (*P* < .05) and better NYHA function classification after the treatment. We also observed fewer cases of deterioration on LAD (*P* = .029) and LVEDD (*P* = .023) in Sacubitril/Valsartan group. In subgroup analysis, our study showed that all 3 kinds of HF patients had better LVEF, LVFS, and NT-proBNP in Sacubitril/Valsartan group (*P* < .05). Our study further indicated that Sacubitril/Valsartan can improve cardiac function and benefit cardiac remolding in aged cancer patients of all 3 kinds of HF. This is the first study to provide new evidence for the use of Sacubitril/Valsartan in aged cancer patients of 3 kinds of HF.

## 1. Introduction

Increased prevalence of cancer and the early detection technologies along with advanced therapies have left a dramatically increasing number of cancer survivors in recent years. In 2022, more than 18 million Americans with cancer were alive, and about half of them have had this diagnosis for more than ten years. It is expected to reach 22.1 million by the start of 2030, and almost two-thirds (67%) of them are 65 years or older aged patients. In mainland China, it is reported that 6.69 million cancer survivors were over 65 years old in 2017, which is going up every year.^[1–3]^

Heart failure (HF) is an illness that consumes substantial healthcare resources, inflicts considerable morbidity and mortality, and significantly affects the quality of life.^[4]^ Cardiovascular disease (CVD), including HF, is believed to have a high morbidity and mortality among cancer patients.^[5–7]^ Such as cancer therapy-related cardio-dysfunction, occurring in approximately 10% of the patients, has the highest mortality of more than 50%.^[8]^ HF in cancer patients often results from all kinds of cardiotoxicity. Advanced antineoplastic therapy nowadays usually has cardiotoxicity, which is believed to be a risk factor of HF.^[5–7]^ What is worse, this kind of cardiotoxicity can also be irreversible and delayed so that cancer patients will have a significantly higher long-term CVD risk and mortality and a poorer cardiac condition in longtime survivorship.^[6,9,10]^ Besides, cancer itself is believed to have a cardiotoxic effect independent of cancer-related therapy.^[11,12]^ Apart from what was said above, age is also a major risk factor of HF. It is reported that Individuals aged ≥65 years take more than 80% of HF deaths, while the aged population is still growing,^[7]^ which means the aging cancer population may suffer more from HF.

Sacubitril/Valsartan (S/V) is a combined formulation of angiotensin receptor inhibitor and neprilysin inhibitor, which inhibits the degradation of natriuretic peptides by neprilysin and brings cardioprotective effects while counteracting the adverse effects of the overactivated renin-angiotensin-aldosterone system (RAAS) including water and sodium retention and vasoconstriction.^[13]^ S/V has been shown to be able to lower the levels of NT-proBNP, improve cardiac function, reverse left ventricular remodeling, decrease cardiovascular disease-related mortality, and improve the life quality in HF patients. Therefore, S/V is currently a first-line recommendation for chronic HF with reduced ejection fraction.^[13–17]^ S/V is the most promising medicine for HF patients with a history of cancer nowadays. Up to now, there have been 3 finished clinical experiments about the use of S/V within cancer patients.^[18–20]^ However, previous studies only paid attention to HfrEF, while HF with preserved ejection fraction (HfpEF) and HF with mildly reduced ejection fraction (HfmrEF) are also problems in the real world, and there is insufficient evidence about its efficacy in aged cancer population with HF.

Although some studies have demonstrated the positive impact of S/V on HfrEF cancer patients, there is still a lack of evidence regarding the drug’s effect on older cancer patients with HfmrEF and HfpEF. Therefore, we conducted this study to investigate the potential benefits of S/V in all 3 types of HF patients with cancer in the elderly population.

## 2. Methodology

### 2.1. Study population

We searched the electronic medical records from January 2016 to February 2022 about aged patients with both cancer and HF diagnoses in our institution. The ones who had received full course treatment with S/V as HF management were selected as the candidates for S/V group, and the ones who had received traditionary HF therapy, including ACEI/ARB or beta-blocker or diuretics without S/V were selected as the control group. The Eligible patients for both groups are those who have received at least 1 echocardiography and the NYHA cardiac function assessment before and 3 months after the initiation of S/V for S/V group or the initiation of traditional HF therapy for the control group; NYHA class should be ≥2 in both groups; age should be ≥65 in both groups; with a history of treated cancer while without current anticancer regimen during the HF therapy period.

### 2.2. Assessment of HF

In both groups, we used the “Criteria from 2021 ESC Guidelines for the diagnosis and treatment of acute and chronic HF^[15]^” to judge the validity of the HF diagnosis. According to the guideline, we divided them into 3 kinds of HF subgroups, including HF with reduced ejection fraction (HfrEF), HF with mildly reduced ejection fraction (HfmrEF), and HF with preserved ejection fraction (HfpEF), by LVEF ≤ 40%, 40% < LVEF < 50%, LVEF ≥ 50%, respectively. The symptoms and signs of HF are necessary for all 3 kinds of HF. For HfpEF, NT-proBNP ≥ 125 pg/mL and/or Objective evidence of cardiac structural and/or functional abnormalities is also needed.^[15]^

### 2.3. Assessment of cancer

The diagnosis of cancer should be based on pathological evidence.

### 2.4. Clinical data collection

The institution where the research is conducted is a large general hospital with standard oncology and cardiovascular departments. Doctors in this hospital recorded the patients’ diagnoses according to the ICD-10 code and all the other information in the electric medical record system. By reviewing the records, medical information on S/V group and control group was obtained, which includes demographic information, clinical characteristics (the type of neoplasms and cancer treatment history), NYHA cardiac function class before and after the initiation and the use of other cardiovascular diseases-related drugs. Data about cardiac biomarkers, NT-proBNP, and Physical examination (blood pressure and heart rate) were also collected if available, along with the collection of echocardiography data at baseline and during HF treatment for up to 9 months.

### 2.5. Statistical analysis

Data are presented as mean ± standard deviation for normal quantitative variables and median [interquartile range] for nonnormal ones. Categorical variables were presented as percentages and compared using the Pearson chi-square test, Wilcoxon signed-rank test, or Fisher precision probability test. Paired sample *t* test and one-way ANOVA were used for comparing continuous variables. We use ordered multinomial logistic regression analysis to analyze the factors that affect patient’s cardiac function. Statistical tests were 2 sided, and we regard 5% as the significance level. All the statistical analyses were performed with SPSS Version 26.

## 3. Result

### 3.1. Basic demographic characteristics

**Figure 1.**
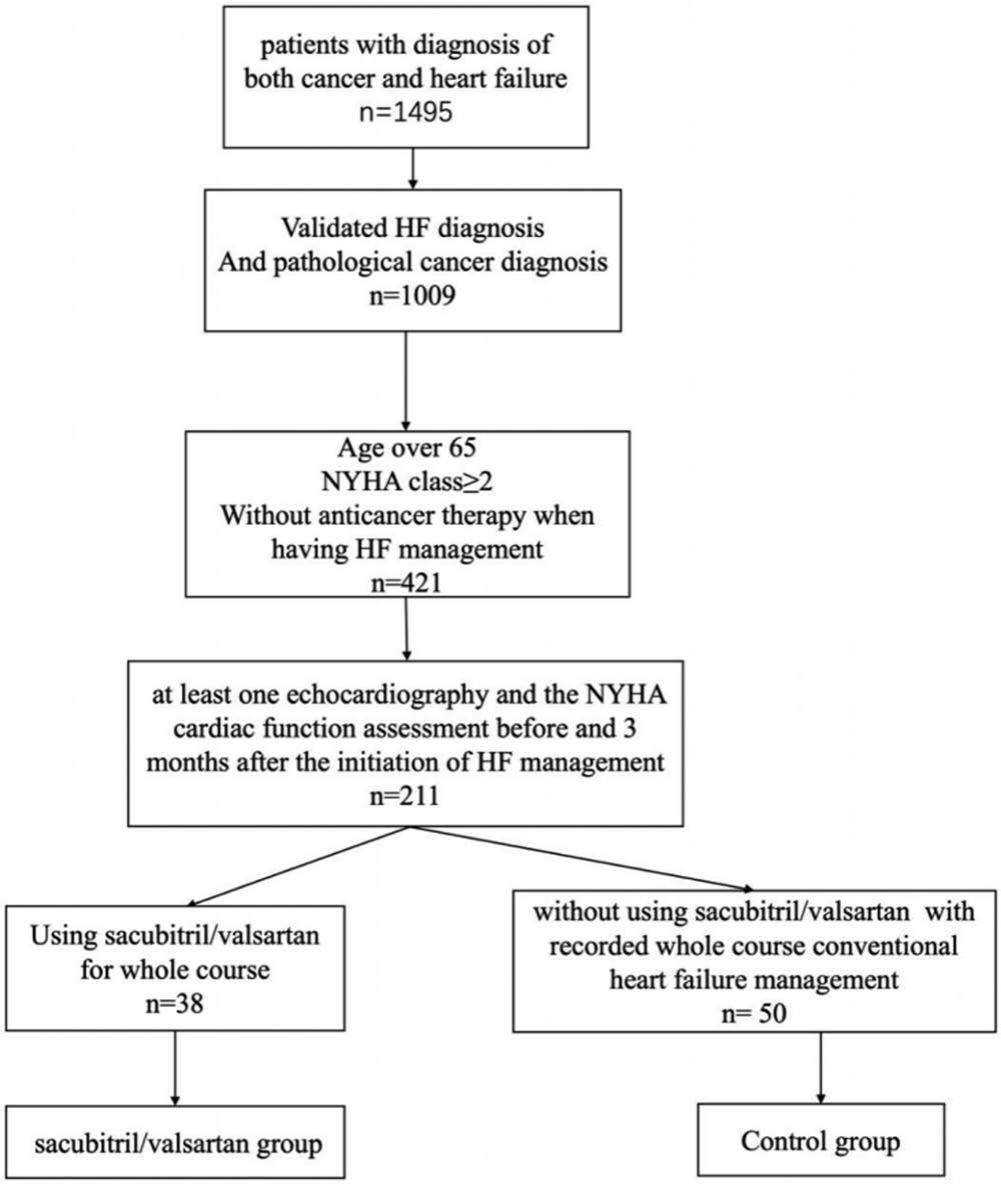
The consort flow diagram of our study. A total of 1495 patients have been diagnosed with both HF and cancer. After the screening, 38 of them were involved in S/V group, while 50 of them were involved in the control group.

We found 1495 patients from the electronic medical record system from January 2016 to February 2022 with the diagnosis of both cancer and HF. After the screening, we filtered 38 patients valid for S/V group and 50 patients valid for the control group (Fig. 1).

The baseline characteristics of both groups are summarized in Table 1. For S/V group, 38 patients were included in this research with a median age of 77.61 ± 8.18, and 23 (60.5%) were male. For the control group, 50 patients were included in this research with a median age of 77.92 ± 7.59, and 32 (64.0%) were male. The S/V group and the control group patients had no difference at the baseline as shown in Table 1. The dosage and frequency of the Sacubitril/Valsartan are 25 mg bid (*n* = 11, 28.95%), 50 mg bid (*n* = 16, 42.11%), and 100 mg bid (*n* = 11, 28.95%) at the beginning. During the HF therapy process, 7 patients changed the dosage. Among them, 2 patients changed from 50 mg bid to 100 mg bid, 2 patients changed from 25 mg bid to 100 mg bid, and 3 patients reduced the dosage from 100 mg bid to 50 mg bid because of low blood pressure; however, no patients had ever given up taking S/V within our observation period and no hypotensive shock or hypotension related adverse event was found within our cases. There were no significant differences in the dose and frequency of S/V used among the 3 different types of HF, HfrEF, HfmrEF, and HfpEF, either at baseline or at the end of observation period (*P* = .344, 0.401).

**Table 1:** Patients baseline characteristics.

Variables	S/V group *N* = 38	Control group *N* = 50	*P* value
Demographic characteristics			
Mean age, y	77.61 ± 8.18	77.92 ± 7.59	.853
Gender, male	23 (60.5%)	32 (64.0%)	.341
Height, cm	159.11 ± 7.15	161.10 ± 5.67	.148
Weight, kg (before)	62.74 ± 10.18	63.83 ± 8.03	.575
BMI, kg/m^2^ (before)	24.82 ± 4.03	24.61 ± 3.00	.424
Weight, kg (after)	62.34 ± 10.41	62.84 ± 8.57	.806
BMI, kg/m^2^ (after)	24.65 ± 4.05	24.22 ± 3.17	.575
Cancer category			*.963*
Lung cancer	17 (44.7%)	20 (40.0%)	
Esophageal cancer	4 (10.5%)	6 (12.0%)	
Hepatic cancer	4 (10.5%)	4 (8.0%)	
Colorectal cancer	4 (10.5%)	8 (16.0%)	
Gastric cancer	2 (5.3%)	4 (8.0%)	
Other	7 (18.4%)	7 (14.0%)	
HF etiology and risk factors			
Hypertension	30 (78.9%)	32 (64.0%)	.128
Atrial fibrillation	16 (42.1%)	28 (56.0%)	.821
COPD	16 (42.1%)	24 (48.0%)	.667
CHD	33 (86.8%)	38 (76.0%)	.062
Diabetes	13 (34.2%)	15 (30.0%)	.674
Smoking	16 (42.1%)	25 (50%)	.462
Drinking	11 (28.9%)	13 (26.0%)	.758
Previous cancer treatment			
Surgery	19 (50.0%)	34 (58.0%)	.087
Chemotherapy	15 (39.5%)	25 (50.0%)	.326
Radiotherapy	7 (18.4%)	8 (16.0%)	.765
Targeted therapy	4 (10.5%)	7 (14.0%)	.751
ICI	2 (5.3%)	3 (6.0%)	.629
Anthracycline	2 (5.3%)	1 (2.0%)	.576
Basic cardiovascular diseases management			
CCB	17 (44.74%)	19 (38.00%)	.524
ACEI/ARB	–	32 (64.00%)	
β-Blocker	29 (76.32%)	37 (74.00%)	.804
Cardiotonic	33 (86.68%)	36 (72.00%)	.804
Diuretic	38 (100.00%)	50 (100.00%)	.094
MRA	36 (94.74%)	45 (90.00%)	.459
SGLT2i	4 (10.53%)	3 (6.00%)	.694
BP and HR			
SBP, mm Hg	130.27 ± 21.57	129.0 ± 17.0	.768
DBP, mm Hg	73.49 ± 12.84	76.2 ± 11.06	.296
HR, bpm	88.70 ± 27.19	86.6 ± 18.84	.676

ARB = angiotensin receptor blocker, BP = blood pressure, ACEI = angiotensin-converting enzyme inhibitors, BMI = body mass index, bpm = beats per minute, CHD = coronary artery heart disease, COPD = chronic obstructive pulmonary disease, DBP = diastolic blood pressure, HR = heart rate, ICI = immune checkpoint inhibitor, MRA = mineralocorticoid receptor antagonist, S/V = Sacubitril/Valsartan, SBP = systolic blood pressure, SGLT2i = sodium-glucose cotransporter-2 inhibitor.

### 3.2. Echocardiographic parameters

**Figure 2.**
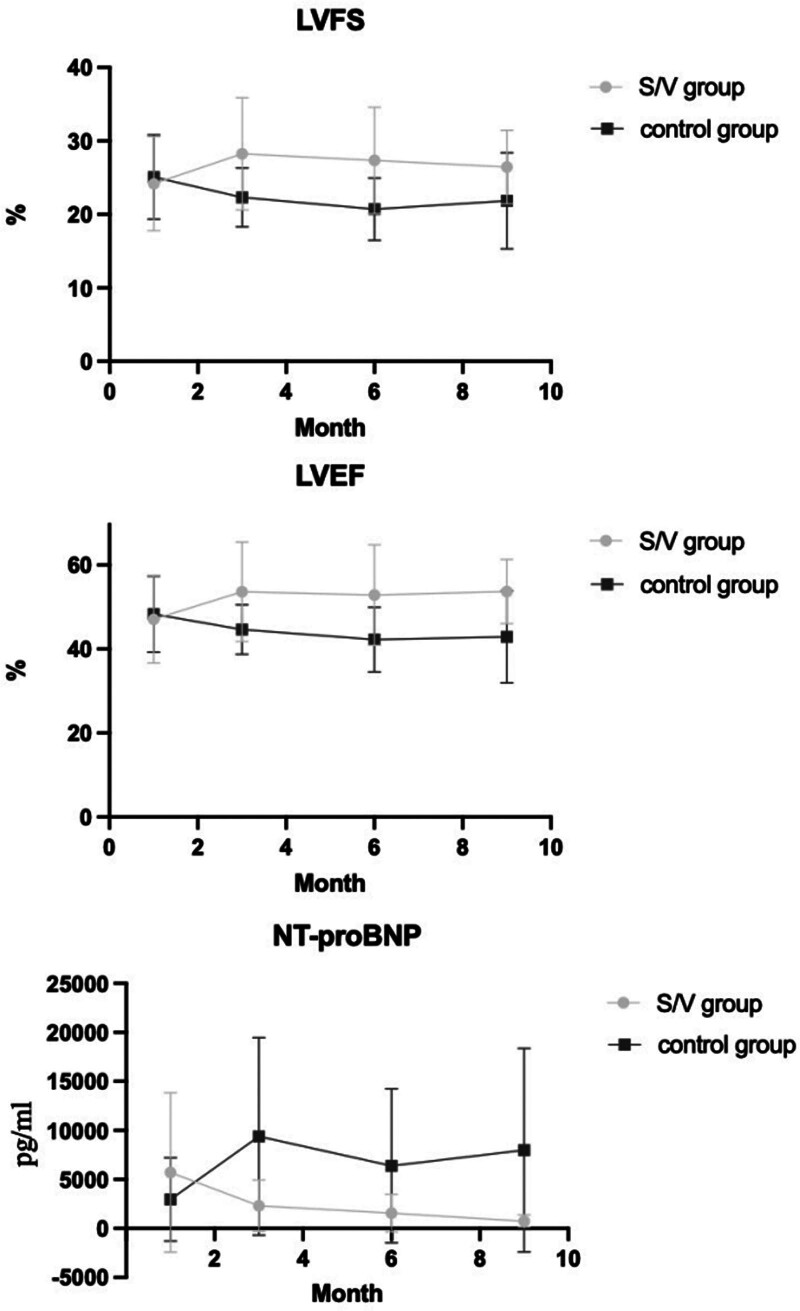
The change in LVEF, NT-proBNP, and LVFS (shown as mean ± SD). After the initiation of HF management, the S/V group showed higher LVFS, LVEF and lower NT-proBNP than control group in 3th, 6th and 9th months. (*P* < .05).

Overall echocardiographic parameters of both groups are shown in Table 2. After treatment, comprised with the control group, S/V group suggested a better cardiac condition; S/V group had higher LVEF in 3rd (*P* = .003), 6th (*P* = .005), 9th (*P* = .000), higher LVFS in 3rd (*P* = .002), 6th (P = .005), 9th (*P* = .008) month; the change on LVEF, LVFS is shown in Fig. 2. Through Analysis of Variance, we also found that the S/V group showed an improvement in Echocardiographic parameters between 1st and 3rd, 6th, and 9th month when compared within the group, whose average LVEF was elevated (*P* = .049, Fig. 2). While the control group showed a deterioration manifested in the reduction of LVEF (*P* = .03), and LVFS (*P* = .02).

**Table 2:** Echocardiographic parameters at the 1st, 3rd, 6th, and 10th month after the initiation of heart failure management.

Variables	Sacubitril/Valsartan group	Control group	*P* value
1st month			
LVFS (%)	24.18 ± 6.41	25.08 ± 5.72	.492
LVEF (%)	47.11 ± 10.45	48.32 ± 9.04	.561
LVEDD (mm)	53.71 ± 6.99	53.04 ± 10.06	.726
LAD (mm)	40.95 ± 7.13	39.96 ± 7.61	.541
3rd month			
LVFS (%)	28.26 ± 7.65	22.32 ± 4.01	**.002**
LVEF (%)	53.65 ± 11.84	44.65 ± 5.91	**.003**
LVEDD (mm)	50.70 ± 9.98	54.67 ± 9.41	.167
LAD (mm)	39.57 ± 8.86	41.78 ± 6.98	.351
6th month			
LVFS (%)	27.33 ± 7.26	20.71 ± 4.24	**.005**
LVEF (%)	52.88 ± 11.94	42.24 ± 7.71	**.005**
LVEDD (mm)	52.19 ± 8.38	56.76 ± 13.33	.227
LAD (mm)	39.50 ± 7.49	42.05 ± 8.45	.347
9th month			
LVFS (%)	26.44 ± 5.01	21.68 ± 6.54	**.008**
LVEF (%)	53.67 ± 7.69	42.90 ± 10.95	**< .001**
LVEDD (mm)	52.83 ± 6.92	54.51 ± 12.65	.589
LAD (mm)	37.89 ± 6.47	40.29 ± 8.82	.303

LAD = left atrium volume diameter, LVEDD = left ventricle end-diastolic diameter, LVEF = left ventricular ejection fraction, LVFS = left ventricular fractional shortening, S/V = Sacubitril/Valsartan.

**Figure 3.**
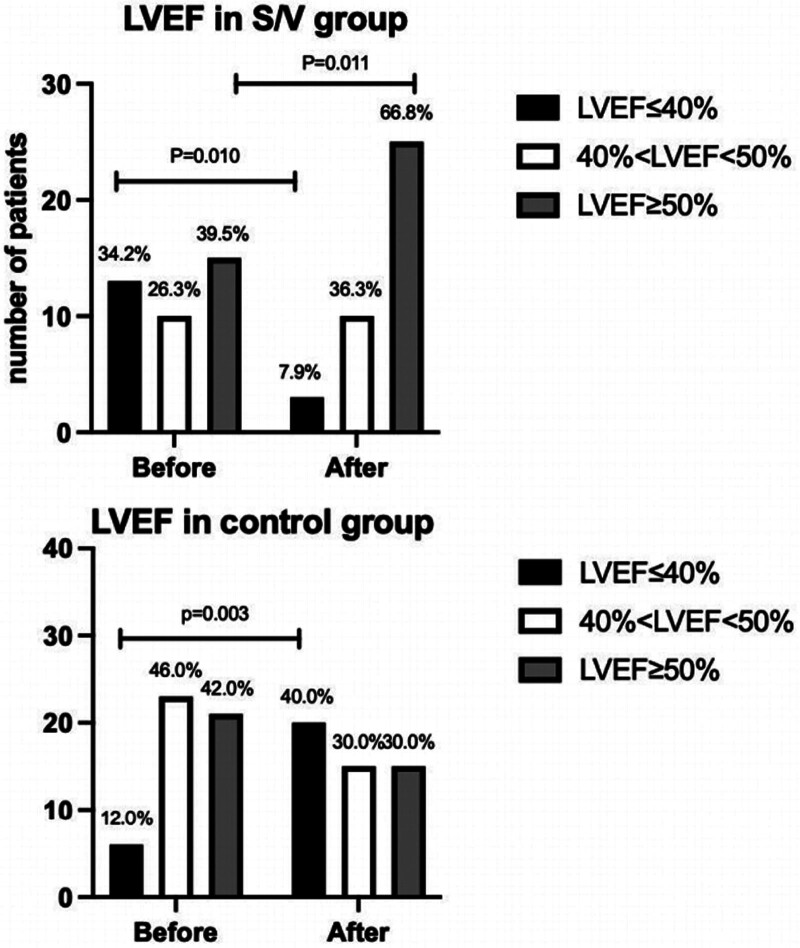
The change in LVEF in the S/V group and control group. After therapy, more patents had a LVEF ≥ 50% and less patients had a LVEF ≤ 40% in S/V group, while in control group more patients had a LVEF ≤ 40%.

We then divided our patients by LVEF into 3 stages: Stage I, severe reduction (LVEF ≤ 40%); Stage 2, middle reduction (40% < LVEF < 50%); Stage 3: preserved LVEF (LVEF ≥ 50%). The number of patients in each stage before and after the initiation of HF management shows in Fig. 2. We found that more S/V group patients had a better LVEF stage, while fewer had changed their LVEF stage into a worse 1 (*P* = .000). Seventeen patients (44.74%) in S/V group and 1 patient (2.00%) in the control group changed from a lower stage into a higher stage. Two patients (5.26%) in S/V group and 19 patients in the control group (38.00%) changed from a higher stage to a lower stage. For S/V group, the most significant elevation in LVEF is 27%, and the greatest decrease decreasing in LVEF is 4%, while in the control group, the most significant elevation in LVEF is 11%, and the greatest reduction in LVEF is 29%. The change in LVEF of both groups shows in Fig. 3.

**Figure 4.**
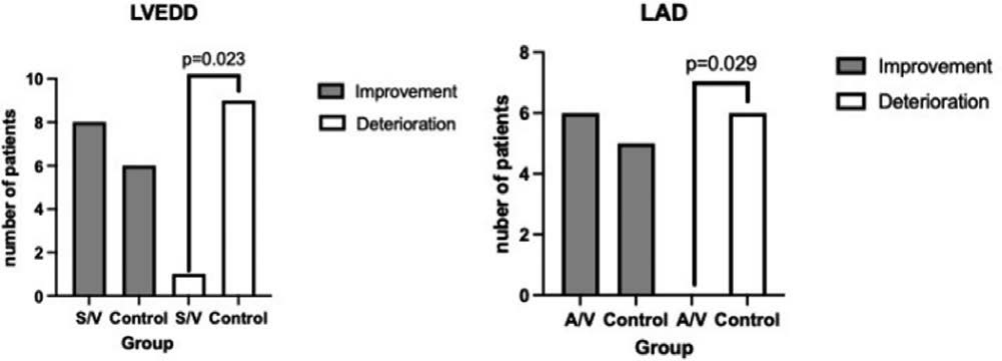
The number of patients with improvement and deterioration on LVEDD and LAD after therapy. After the initiation of HF management, S/V group had less deteriorated LVEDD and LAD cases than control group (*P* < .05).

Our study used 53 mm for women and 52 mm for men as the upper reference value of LVEDD, 37 mm for women, and 43 mm for men as the upper reference value for LAD, which are set for the east China aged population.^[21]^ Then we divided patients into subgroups by normal and abnormal LVEDD and LAD. If the patients had changed from abnormal to normal, then we regarded it as an improvement. At the same time, if a patient had changed from normal to abnormal, then we regarded it as deterioration. The number of improvements and deterioration of LVEDD and LAD in both groups is shown in Fig. 4. We observed fewer patients had deteriorated in both LVEDD and LAD. In S/V group, only 1 patient deteriorated in LVEDD, compared with the control group, which is 9 (*P* = .023). In S/V group, no patient showed deterioration in LAD, compared with the control group, which is 6 patients (*P* = .029).

### 3.3. Cardiac biomarker

Most of the patients included in this study underwent testing of cardiac biomarkers, including NT-proBNP, CK-MB, MYO, and c-Tnt. The main outcome is shown in Table 3. Compared between groups, S/V group had a lower NT-proBNP in the 3rd (*P* = .04), 6th (*P* = .015), and 9th (*P* = .000) month (Fig. 2). Other indicators showed no significant difference, whether in vertical comparison (compared within the group between each month) or horizontal comparison (compared between 2 groups at each month). Compared within groups between each month, NT-proBNP decreased (*P* = .004) in S/V group, while in the control group, NT-proBNP was elevated (*P* = .009).

**Table 3:** Cardiac biomarkers in the 1st, 3rd, 6th, and 9th month after the initiation of heart failure management.

Variables	S/V group	Control group	*P* value
1st month			
MYO (ng/mL)	138.83 ± 188.61	77.50 ± 98.17	.128
CK-MB (ng/mL)	7.89 ± 10.89	126.49 ± 129.36	.407
c-Tnt (ng/mL)	0.38 ± 0.99	0.21 ± 0.36	.445
NT-proBNP (pg/mL)	5721.31 ± 8133.29	2963.28 ± 4248.86	.065
3rd month			
MYO (ng/mL)	103.77 ± 145.73	99.84 ± 124.48	.928
CK-MB (ng/mL)	4.98 ± 8.64	5.88 ± 6.17	.704
c-Tnt (ng/mL)	0.26 ± 0.56	0.029 ± 0.029	.160
NT-proBNP (pg/mL)	2309.68 ± 2631.66	9394.99 ± 10087.42	**.004**
6th month			
MYO (ng/mL)	113.95 ± 225.01	93.32 ± 102.68	.756
CK-MB (ng/mL)	3.66 ± 102.68	5.26 ± 6.13	.400
c-Tnt (ng/mL)	0.043 ± 0.026	0.078 ± 0.11	.429
NT-proBNP (pg/mL)	1574.90 ± 1937.17	6392.20 ± 7845.78	**.015**
9th month			
MYO (ng/mL)	67.70 ± 97.56	98.14 ± 105.67	.351
CK-MB (ng/mL)	5.41 ± 6.76	21.63 ± 61.01	.282
c-Tnt (ng/mL)	0.20 ± 0.37	0.084 ± 0.12	.198
NT-proBNP (pg/mL)	739.54 ± 640.88	7982.18 ± 1037.00	**< .001**

CK-MB = creatine kinase-MB, MYO = myoglobin, NT-proBNP = N terminal pro B type natriuretic peptide, S/V = Sacubitril/Valsartan.

### 3.4. Within-group comparison

We used one-way ANOVA to test the changes of various indicators between the S/V group and the control group before treatment, and at 3, 6, and 9 months after treatment. For the S/V group, MYO, CK-MB, c-Tnt, SBP, DBP, HR, FS, LVEF, LVEDD, and LAD showed no statistical significance (*P* > .05). The change in NT-proBNP was statistically significant (*P* = .001), with a significant decrease observed at 6 months (*P* = .029) and 9 months (*P* = .004) after treatment compared to before treatment. In the control group, MYO, CK-MB, c-Tnt, SBP, DBP, HR, LVEDD, and LAD showed no statistical significance (*P* > .05). However, NT-proBNP (*P* = .003), LVEF (*P* = .018), and LVFS (*P* = .005) showed statistical significance. Among them, the NT-proBNP level was significantly increased at 3 months (*P* = .049) and 9 months (*P* = .034) compared to before treatment; the LVEF level was significantly decreased at 6 months (*P* = .036) compared to before treatment. The LVFS level was significantly decreased at 6 months (*P* = .005) compared to before therapy.

### 3.5. Clinical manifestation

We use NYHA stages to judge the clinical manifestation of HF. As illustrated in Table 4, the difference is shown between groups after the treatment (*P* = .001), which was more patients had a lower stage in S/V group and fewer patients had a higher stage. At the same time, it is inverse in the control group. The change in the NYHA stage between groups also showed a difference (*P* = .001); we can see more patients in the S/V group had improvement in the NYHA stage, and fewer patients had deterioration. There is no statistical difference in BP and HR in the horizontal comparison and vertical comparison of both groups (*P* > .05).

**Table 4:** The NYHA functional classification before and after the initiation of heart failure management.

	S/V group (n)	Control group (n)	*P* value
Before			
Stage II	4	12	.588
Stage III	26	26	
Stage IV	8	12	
After			**.001**
Stage II	15	4	
Stage III	18	30	
Stage IV	5	16	
The change of heart function			
Deterioration	4	20	**.001**
Improving	16	7	
Stabilization	18	23	

NYHA = New York Heart Association function classification, S/V = Sacubitril/Valsartan.

### 3.6. Factors affecting patient’s cardiac function

We used NYHA classification to define the patient’s cardiac function status. Before and after medication, if a patient’s NYHA stage decreased, it was defined as an improvement in cardiac function; if the NYHA stage increased, it was defined as deterioration, and if the NYHA stage remained the same, it was defined as stabilization. The changes in the patient’s cardiac function are shown in Table 4. We used ordered multinomial logistic regression analysis to analyze the factors that affect the changes in the patient’s cardiac function. The included indicators were NT-proBNP, SBP, DBP, HR, FS, EF, LV, LA before treatment; gender, age, weight, and BMI before and after treatment; whether to use diuretics intravenously, whether there is hypertension, whether there is atrial fibrillation, whether there is diabetes, whether there is coronary heart disease, whether there is COPD, whether there is a history of smoking and drinking, HF management methods, past tumor treatment methods, and cancer types. Among them, LVFS before medication (*P* = .029, OR [95% CI] = 1.62 [1.05, 2.49]), LVEF before medication (*P* = .021, OR [95% CI] = 0.73 [0.56, 0.96]), LAD before medication (*P* = .022, OR [95% CI] = 0.90 [0.82, 0.98]), the use of S/V (*P* = .022, OR [95% CI]=0.02 [0.00, 0.57]), surgery (*P* = .028, OR [95% CI] = 4.10 [1.17, 14.39]), and lung cancer (*P* = .035, OR [95% CI] = 0.07 [0.01, 0.84]) were related to the change of the patient’s cardiac function after treatment (Table 5).

**Table 5:** Multinomial logistic regression analysis of the association between factors and the changes in the patient’s cardiac function after therapy.

Indicators	*P*	OR (95% CI)
LVFS	.029	1.62 (1.05, 2.49)
LVEF	.021	0.73 (0.56, 0.96)
LAD	.022	0.90 (0.82, 0.98)
Using S/V	.022	0.02 (0.00, 0.57)
Surgery	.028	4.10 (1.17, 14.39)
Lung Cancer	.035	0.07 (0.01, 0.84)

LVEF = left ventricular ejection fraction, LVFS = left ventricular fractional shortening; LAD = left atrium volume diameter, S/V = Sacubitril/Valsartan.

### 3.7. The effect of S/V in different HF

**Figure 5.**
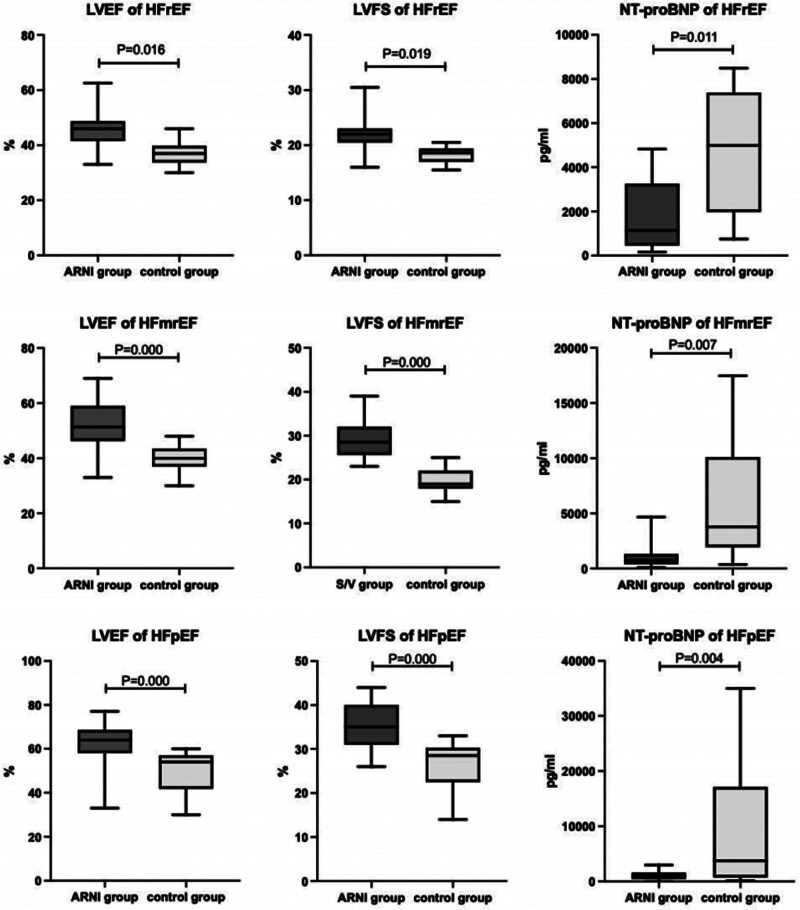
LVFS, LVEF, NT-proBNP in hFrEF, hFmrEF, and hFpEF, respectively, compared between S/V and control groups after treatment (*P* < .05). After treatment, the S/V group had better results than the control group in terms of LVFS, LVEF, and NT-proBNP in all 3 types of HF: hFrEF, hFmrEF, and hFpEF (*P* < .05).

We divided S/V group and control group patients into subgroups by LVEF. For S/V group, there are 13 HfrEF patients, 10 HfmrEF patients, and 15 HfpEF patients. For the control group, there are 6 HfrEF patients, 23 HfmrEF patients, and 21 HfpEF patients. All the result is shown in Table 6. At the baseline, there is no difference between the S/V group and the control group within these subgroups (*P* > .05). However, there is a better heart function showed in S/V group within all 3 kinds of HF subgroups (Fig. 5).

**Table 6:** LVFS, LVEF, NT-proBNP in HFrEF, HFmrEF, HFpEF after treatment in Sacubitril/Valsartan (S/V) and control groups.

	S/V group	Control group	*P* value
HFrEF	*n* = 13	*n* = 6	
Before			
NT-proBNP (pg/mL)	6610.42 ± 9600.73	1385.00 ± 1249.29	.251
LVEF (%)	36.08 ± 4.43	33.67 ± 3.33	.254
LVFS (%)	17.85 ± 2.56	16.33 ± 2.25	.234
LAD (mm)	42.38 ± 6.80	44.33 ± 5.65	.551
After			
NT-proBNP (pg/mL)	1872.86 ± 1601.49	4770.25 ± 2884.21	**.011**
LVEF (%)	46.72 ± 8.01	37.11 ± 5.18	**.016**
LVFS (%)	22.04 ± 3.32	18.28 ± 1.67	**.019**
LAD (mm)	40.40 ± 8.57	40.78 ± 3.84	.569
HFmrEF	*n* = 10	*n* = 23	
Before			
NT-proBNP (pg/mL)	7948.25 ± 9633.17	3563.88 ± 3312.73	.074
LVEF (%)	45.20 ± 2.94	43.65 ± 2.55	.136
LVFS (%)	22.50 ± 3.01	22.13 ± 1.77	.664
LAD (mm)	41.35 ± 7.25	40.50 ± 4.01	.732
After			
NT-proBNP (pg/mL)	1163.73 ± 1325.14	6036.05 ± 5187.25	**.007**
LVEF (%)	55.37 ± 8.119	40.64 ± 3.782	**< .001**
LVFS (%)	28.92 ± 4.59	19.95 ± 2.47	**< .001**
LAD (mm)	37.40 ± 5.52	4.83 ± 9.42	.294
HFpEF	*n* = 15	*n* = 21	
Before			
NT-proBNP (pg/mL)	3466.13 ± 5141.83	2755.25 ± 5141.82	.695
LVEF (%)	57.93 ± 4.99	57.62 ± 3.17	.993
LVFS (%)	30.80 ± 3.23	30.81 ± 2.86	.819
LAD (mm)	40.00 ± 8.81	37.19 ± 7.76	.391
After			
NT-proBNP (pg/mL)	1078.40 ± 841.13	8412.85 ± 9868.82	**.004**
LVEF (%)	64.03 ± 7.23	50.66 ± 9.02	**< .001**
LVFS (%)	34.63 ± 5.30	26.45 ± 7.25	**< .001**
LAD (mm)	38.03 ± 9.77	38.44 ± 8.22	.892

LAD = left atrial diameter, LVEF = left ventricular ejection fraction, LVFS = left ventricular fractional shortening, NT-proBNP = N terminal pro B type natriuretic peptide, S/V = Sacubitril/Valsartan.

## 4. Discussion

To our knowledge, this is the first study paying attention to the effect of S/V in aged cancer patients of all 3 kinds of HF. We observed that S/V has the ability to improve cardiac function and benefit the cardiac remolding in not only HfrEF but also HfmrEF and HfpEF aged patients with cancer, manifested in higher LVEF, LVFS, and lower NT-proBNP in both whole group analysis and subgroup analysis (Figs. 2 and 5). NYHA functional classification and fewer patients had deteriorated in both LVEDD and LAD in S/V group compared with the control group were also observed (Table 4; Fig. 4).

The previous studies are in line with our result, showing the cardiac protection effect of S/V on cancer patients with HF. The study of Ana Martín-Garcia etc, carried out in Spanish, showed S/V had greatly improved the LVEF from 33% to 42% and reduced the NT-proBNP (*P* < .001) in patients with HfrEF due to cancer therapy along with the reverse of cardiac remodeling benefit, which is believed to be 1 of the most critical effects of S/V and is related to the reduction in the combined end point of death or hospitalization for HF patients.^[22]^ Studies carried on in India and Austria taking in cancer patients with HF with an average LVEF of 26.7 ± 5.4% and 26.8 ± 5.4, respectively, also showed the beneficial effects of S/V on left ventricular remodeling, diastolic dysfunction, and clinical manifestation within patients with cancer patients with HF. Although all the studies above showed the promising effect of S/V in the oncology area to manage HF among cancer patients, they focused only on HfrEF. However cancer was believed to be twice as frequent as a cause of death in HfpEF and HfmrEF versus HfrEF.^[23]^ It is well established that S/V is the top 1 recommendation for HfrEF, while there is limited evidence that limits the use of S/V among patients with HfmrEF and HfpEF. Our study paid attention to the aged cancer population with all 3 kinds of HF in the real world, and it not only likewise found the beneficial effect of S/V in the aged cancer population with HfrEF but also did our study found its heart-protective role in aged cancer population of HfmrEF and HfpEF. There are already some studies that support the effective role of S/V in HfpEF and HfmrEF within normal patients. A meta-analysis of Jianbin Qin, etc, taking in 13 articles, demonstrated that HfmrEF patients had better cardiac function manifested in better echocardiographic parameters and cardiac parameters after using S/V, including higher LVEF, LVFS, and lower NT-proBNP.^[24]^ Another study carried in HfpEF patients found S/V group had a reduction in NT proBNP, LA volume, and improvement in NHYA class compared to Valsartan.^[25]^

Our study also found that the control group showed a deterioration in heart function even after initiation of conventional HF management, which may result from the following reasons: the delayed cardiotoxicity of cancer therapy; older age is related to poorer heart function; the conventional HF therapy in our control group, which is in line with the current clinical situation in China, may not be enough for heart protection among this group of patients.^[26]^ In previous studies about other causes of HF, S/V was already believed to be superior to conventional HF drugs such as ACEI/ARB, beta-blockers, and MRAs.^[13,27,28]^ The advantage of S/V is also found in the aged population, even with drug discontinuation accounting for real-world.^[29]^ Our study in S/V group also showed the same result, finding S/V is superior to conventional HF therapy in real-world aged cancer population with all 3 kinds of HF.

The reason why S/V is superior to beta-blockers, ACEI, and MRAs mainly because of the beneficial effects of neprilysin inhibition resulting in reducing myocardial fibrosis and improving cardiac remodeling by relieving wall stress, inflammation, hypertrophy, and cell death. Its anti-arrhythmic effect through sympathetic inhibition and the increase of enkephalins, endorphins, and bradykinin are also specially advantageous to HF.^[13]^ Apart from all these above, S/V is believed to be able to attenuate oxidative stress by reducing the production of reactive oxygen species (ROS) and malonyl dialdehyde (MDA), which may also be an important hidden mechanism of the beneficial effect.^[30]^ Oxidative stress is detrimental to cardiac function and structure, playing an essential role in the pathophysiology of cardiac remodeling, interstitial fibrosis, and HF.^[31]^ At the same time, oxidative stress is also believed to be higher in cancer patients.^[32]^ Besides, the cardiotoxic antineoplastic therapy will also increase the oxidative burden of cancer patients and lead to cardiotoxicity, and age also has been associated with the production of high levels of ROS.^[33–36]^ Oxidative stress is also related to mitochondria function, which plays a vital role in cellular apoptosis and heart dysfunction, while reducing oxidative stress is believed to have cardiac protection.^[36–39]^

### 4.1. Limitation

First, we did not get data about the effect S/V has on diastolic dysfunction and right ventricular function, yet they are also important parts of cardiac function; further study is needed for them. Second, the use of S/V also seems promising in the aged cancer population with all 3 kinds of HF to the evidence in this limited group of patients. However, since this study is an observational study in a small number of cases, a large-scale prospective study should be carried out in a larger population to prove the causal relationship between using this drug and improvement in cardiac function among HF patients with cancer history. Besides, even our study suggested S/V is safe and useful in clinical practice among this group of patients. Nevertheless, we did not reach a better understanding of its possible adverse effects; more evidence is required to prove its safety and bring us a comprehensive understanding.

### 4.2. Conclusion

Up to now, previous studies have only paid attention to hFrEF, while hFpEF and hFmrEF are also problems in the real world, and there is insufficient data about its efficacy in the aged cancer population. Our study is the first study to pay attention to aged cancer patients with all 3 kinds of HF. Our study showed that S/V might have the ability to improve cardiac function and benefit the cardiac remolding in this group of people, manifested in higher LVEF and LVFS, lower NT-proBNP, NYHA functional classification, and relatively more favorable progression on LVEDD and LAD compared with the control group. Our study further indicated that S/V has the ability to improve cardiac function and benefit the cardiac remolding in aged cancer patients of all 3 kinds of HF, and first provide new evidence for using S/V among aged cancer patients of all 3 kinds of HF. More potentials of S/V are still waiting for us to unearth.

## Acknowledgments

Thanks to Dr Kanghua Lu, Dr Zhilan Lu, Xinwu Chen, and Dr Rui Lan for helping with this study.

## Author contributions

**Conceptualization:** Zhulu Chen, Yuxi Zhu, Diansa Gao, Zhong Zuo.

**Data curation:** Zhulu Chen, Chuan Zhang, Min Mao.

**Investigation:** Zhulu Chen, Min Mao.

**Writing—original draft:** Zhulu Chen, Chuan Zhang.

**Methodology:** Chuan Zhang, Yuxi Zhu, Diansa Gao, Min Mao, Zhong Zuo.

**Supervision:** Chuan Zhang, Diansa Gao, Zhong Zuo.

**Resources:** Yuxi Zhu, Min Mao, Zhong Zuo.

**Validation:** Yuxi Zhu, Diansa Gao.

**Writing—review & editing:** Yuxi Zhu, Diansa Gao, Zhong Zuo.

**Project administration:** Diansa Gao, Min Mao, Zhong Zuo.

**Funding acquisition:** Zhong Zuo.
